# Mesonephric-like adenocarcinoma of the uterine corpus with focal sarcomatous differentiation: A case report

**DOI:** 10.20407/fmj.2024-017

**Published:** 2024-10-31

**Authors:** Serika Kanao, Makoto Urano, Kazuhisa Fujita, Fumi Utsumi, Kazuhiro Sugihara, Kiyosumi Shibata

**Affiliations:** 1 Department of Obstetrics and Gynecology, Fujita Health University Bantane Hospital, Nagoya, Aichi, Japan; 2 Department of Diagnostic Pathology, Fujita Health University Bantane Hospital, Nagoya, Aichi, Japan

**Keywords:** Mesonephric-like adenocarcinoma, Uterine corpus cancer, Sarcoma

## Abstract

Mesonephric-like adenocarcinoma of the uterine corpus was first reported in 2016, and added as a new item to the 5th edition of the WHO classification in 2020. It accounts for approximately 1% of patients with uterine corpus cancer, being rare. The histology of this carcinoma varies, making pathological diagnosis difficult. A diagnosis is made at an advanced stage in comparison with other histological types of uterine corpus cancer, and the prognosis is reportedly poor with high-level malignancy. In addition, there are few case reports of mesonephric-like adenocarcinoma of the uterine corpus with sarcoma. In this study, we report a patient in whom preoperative chemotherapy was performed under a diagnosis of cervical carcinoma, but the resected specimen led to a diagnosis of sarcomatous component-mixed mesonephric-like adenocarcinoma of the uterine corpus after surgery.

## Case

Patient: A 65-year-old female (gravida 0, para 0). The age at the time of menopause was 50 years.

Present illness: There was no symptom. On a health checkup at a local clinic, chest X-ray showed multiple nodules in the lung fields. For detailed examination, thoracic-pelvic computed tomography (CT) was performed. Uterine enlargement and multiple lung tumors were detected, suggesting a primary malignant uterine tumor. She was referred to our hospital for detailed examination.

Medical history: Benign goiter (surgery in her 20s), hypertension.

Family history: Not contributory.

Findings on examination: Macroscopically, the uterine cervix was replaced by the tumor. Internal examination showed the adult-head-sized uterus and poor mobility, suggesting left parametrium invasion.

**Figure 1:**
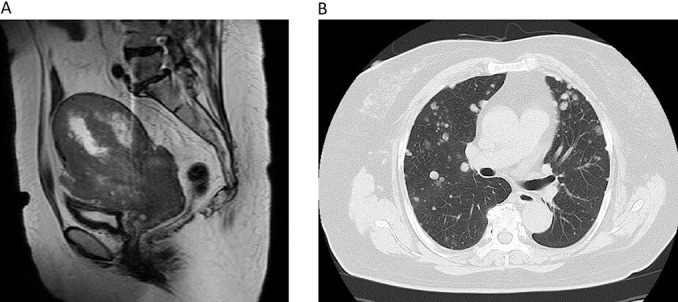
A: Plain MR image of the pelvis before treatment (T2-weighted image): A low-signal-intensity tumor occupying the uterine cervix to corpus was observed. In the inner area of the tumor, hemorrhage was noted. On the anterior wall side, infiltration beyond the serous membrane was suspected. B: Thoracic CT image: In the bilateral lung fields, masses suggestive of multiple metastases were observed.

Contrast-enhanced magnetic resonance imaging (MRI) of the pelvis: The tumor involved the uterine cervix to corpus, measuring approximately 15×8.5×10 cm. Both T1- and T2-weighted images of the tumor showed an uneven low signal intensity. In the inner area, hemorrhage was partially noted. On the anterior wall side, infiltration beyond the serous membrane was suggested (Figure 1-A).

Contrast-enhanced CT of the thorax-pelvis: Multiple lung metastases (Figure 1-B) were detected. There was no other distant metastasis.

Concerning tumor markers, the SCC, CA125, CA19-9, and carcinoembryonic antigen (CEA) levels were 0.7 ng/mL, 30.3 U/mL, 2.6 U/mL, and <1.7 ng/mL, respectively, being within the normal ranges.

**Figure 2:**
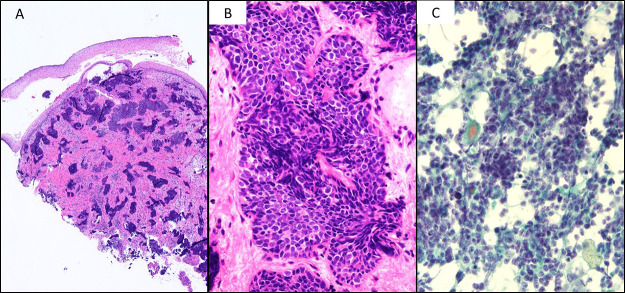
Preoperative pathological findings A: Cervical biopsy (HE staining, ×4) There was no atypia of the covering stratified squamous epithelium. In the deep layer, a large number of tumor nests were observed like small clusters to diffusely. B: Cervical biopsy (HE staining, ×20) Crushed bare-nucleus-like cells with a high N/C ratio and rough nuclear chromatin showed alveolar, solid sheet-like proliferation. C: Cytodiagnosis of the endometrium (Papanicolaou staining, ×40) As described for the cervical specimen, overlapping clusters of bare nucleus-like cells with a high N/C ratio were observed.

Histological diagnosis of the uterine cervix: There was no atypia of the covering stratified squamous epithelium of the superficial layer, but a large number of tumor nests consisting of bare nucleus-like atypical cells diffusely distributed from small clusters, with a high N/C ratio, were observed in the deep layer (Figure 2-A). Tumor cells showed a rough chromatin pattern (Figure 2-B). On immunostaining, a p16-negative reaction was noted, but the specimen was partially positive for synaptophysin, suggesting small cell neuroendocrine carcinoma.

Cytodiagnosis of the endometrium: A bare nucleus-like cell population with a high N/C ratio was observed, as described for the cervical tissue specimen (Figure 2-C).

As the tumor involved the uterine cervix to corpus, it was difficult to identify a primary site. However, assuming small cell neuroendocrine carcinoma, the possibility of a primary cervical tumor was considered to be high, and chemotherapy was performed under a diagnosis of stage IVB cervical carcinoma at the start of treatment.

Treatment course: A total of three courses of TC therapy (PTX: 175 mg/m^2^, CBDCA: AUC6) were performed. CT confirmed slight reductions in the primary focus and multiple lung shadows; the patient was regarded as achieving a partial response (PR). TC therapy was continued, and a total of 7 courses were performed. CT showed a marked reduction in the primary focus and the disappearance of multiple lung lesions, which could not be identified. Imaging findings confirmed tumor reduction, and internal examination findings suggested that surgical extirpation is possible. Considering a histological diagnosis of a small cell neuroendocrine tumor on biopsy, laparotomy was performed.

**Figure 3:**
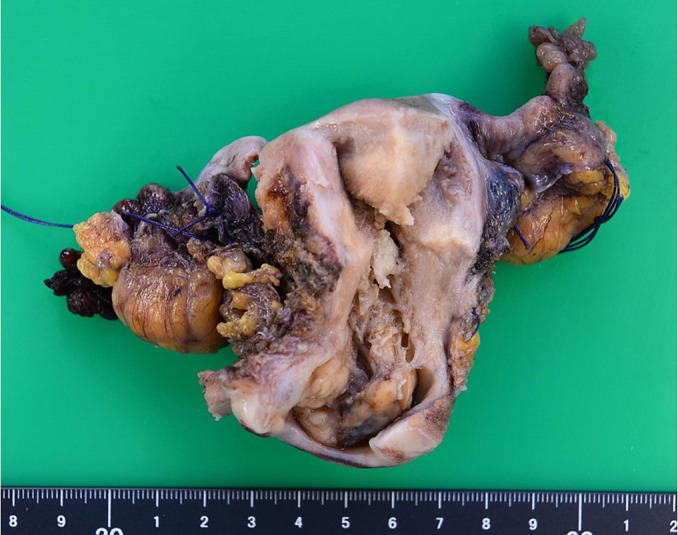
Macroscopic findings of the extirpated uterus and bilateral appendages A tumor involving the uterine cervix to corpus and measuring approximately 4×4 cm was observed.

Surgical findings: Under general anesthesia, median incision of the lower abdomen was performed for laparotomy. The uterus was about the size of a fist. The bilateral appendages were macroscopically normal. There was no ascites. Pretreatment images showed infiltration to the vesico-uterine pouch peritoneum, but this site had become scarred, and adhesion was observed. However, exfoliation was possible. There was no other residual intraperitoneal tumor, and modified radical hysterectomy and bilateral salpingo-oophorectomy were performed. Slight swelling of the left obturator lymph node was observed, and it was extirpated for biopsy. The duration of surgery was 2 hours and 35 minutes, and the blood loss volume was 200 mL. There was no intraoperative complication, and surgery was completed. The uterus measured 5×8×3 cm in diameter. The tumor diameter was 4×4 cm, and the tumor was consecutively located in the uterine cervix to corpus (Figure 3).

**Figure 4:**
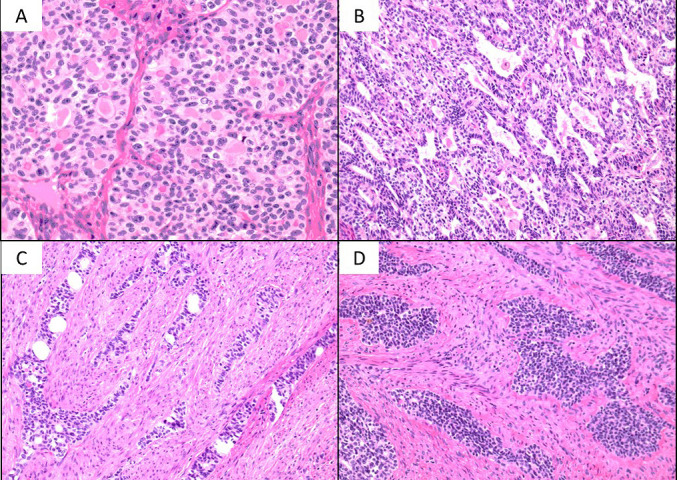
Histopathological findings of the extirpated uterus A: Eosinophilic substance-containing fused duct-like proliferation (HE staining, ×20) B: Fissure-like to irregular duct-like proliferation (HE staining, ×20) C: Cord-like proliferation (HE staining, ×20) D: Solid alveolar proliferation (HE staining, ×20)

**Figure 5:**
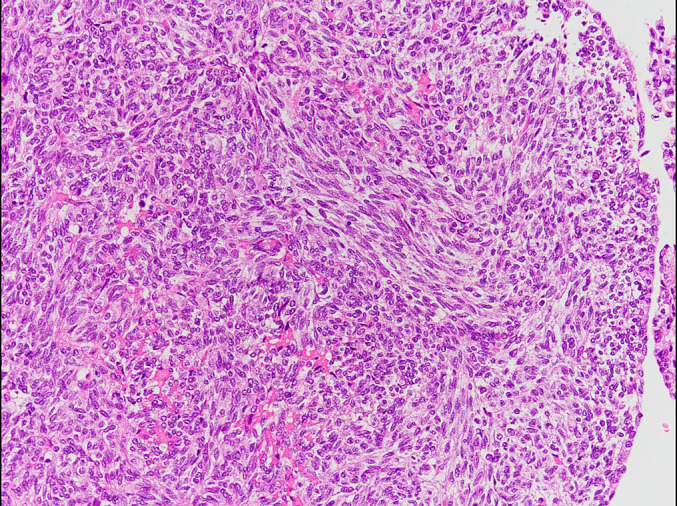
Histopathological findings of the extirpated uterus (HE staining, ×20) A sarcomatous area consisting of fascicular proliferation of atypical spindle cells

**Figure 6:**
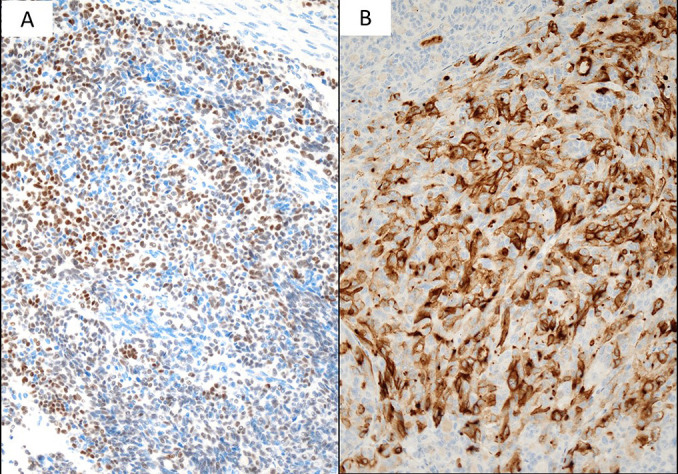
Immunostaining A: Positive for GATA3 (×20) B: Positive for CD10 (×20)

Histology of surgical materials: In the tumor area, round to polygonal nuclear cells showed various carcinoma features: eosinophilic substance-containing fused duct-like (Figure 4-A), fissure-like to irregular duct-like (Figure 4-B), and sparse ductal structure-containing cord-like (Figure 4-C) or solid alveolar (Figure 4-D) structures. Furthermore, partially, a sarcomatous area with fascicular proliferation of atypical spindle cells was observed (Figure 5). As a background factor, the residual existing endometrial gland was not clear. On immunostaining, tumor cells were positive for GATA3 (Figure 6-A), CD10 (Figure 6-B), calretinin, TTF-1, and Pax-8 at various degrees, but were negative for ER.

Based on these findings, no mesonephric remnant was confirmed as a background factor. A preoperative cervical biopsy specimen did not show malignancy in the covering squamous epithelium. Tumor growth was present below the epithelium, and the resected specimen demonstrated that, macroscopically/histologically, the tumor primarily involved the endometrium on the uterine corpus side rather than the uterine cervix, suggesting that the lesion locus is the uterine corpus. A diagnosis of primary mesonephric-like adenocarcinoma of the uterine corpus was made.

**Figure 7:**
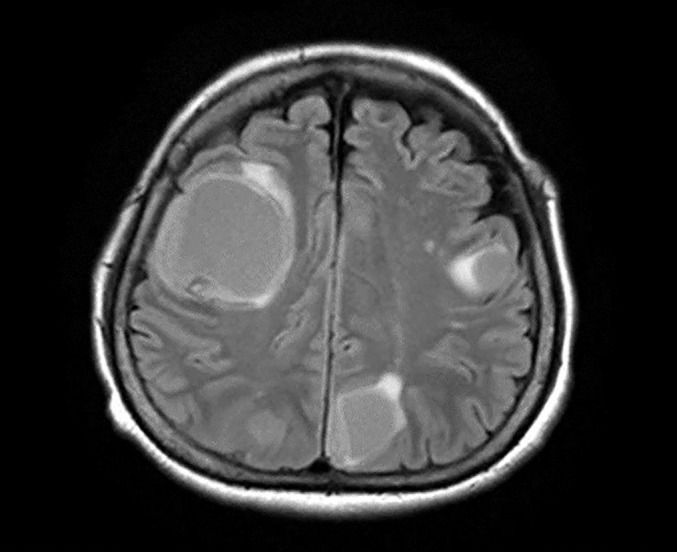
T2-weighted cerebral MRI revealed high-signal-intensity lesions with cysts in the bilateral cerebral hemispheres.

Furthermore, a reduction in GATA3 expression or its disappearance was observed in a portion of the tumor, an area with spindle cell proliferation, suggesting sarcomatous differentiation (Figure 5). In the extirpated tissue, solid proliferation of bare nucleus-like cells with a high N/C ratio, which resembles a biopsy finding suggestive of small cell neuroendocrine carcinoma, was primarily observed on the cervical side, and slight synaptophysin expression was noted. Therefore, the poorly differentiated component of this tumor may have been evaluated. There was no metastasis in any extirpated lymph node specimen. Although preoperative imaging showed chemotherapy-related tumor reduction, degeneration of tumor cells was not marked in the resected specimen, suggesting the appearance of resistance to chemotherapy. The above pathological results led to a final diagnosis of uterine corpus cancer (mesonephric-like adenocarcinoma) ypT2N0M1, Stage IVB. The postoperative course was favorable, and the patient was discharged 12 days after surgery. She did not wish to undergo additional treatment, and a strategy to continue follow-up without performing postoperative adjuvant therapy was adopted. She continued to consult the outpatient clinic, but was brought to our hospital by ambulance with speech disorder and gait disorder 17 weeks after surgery. Contrast-enhanced cerebral MRI revealed multiple high-signal-intensity masses with cysts in the white matter of the bilateral cerebral hemispheres on T2-weighted images, suggesting multiple metastases (Figure 7). Under general anesthesia, biopsy of the cerebral lesions was performed. Findings similar to the uterine tumor, as described above, were observed, and treatment with a γ-knife is being performed under a diagnosis of metastases.

## Discussion

Mesonephric adenocarcinoma (MA) may develop through the mesonephric remnant. On the other hand, carcinoma that has the histological characteristics of MA and an MA-like structure, but not the mesonephric remnant or hyperplasia as a background factor is termed mesonephric-like adenocarcinoma (MLA).^1,2^ MLA of the uterine corpus was first reported in 2016, and added as a tumor of the uterine corpus to the 5th edition of the WHO classification in 2020.^1–3^

MLA develops in the uterine corpus and ovary, showing various proliferation patterns. In the inner cavity, eosinophilic secretion is contained, and this carcinoma is defined as a tumor that sometimes has a characteristic nucleus resembling the nucleus of papillary thyroid carcinoma.^1^ MA may develop through the mesonephric (Wolffian duct) remnant. However, MLA has histological characteristics similar to those of MA in the absence of the mesonephric remnant; its pathogenesis has been discussed. Currently, it is often complicated by endometriosis or Mullerian tumor, such as borderline malignant tumors, poorly differentiated serous carcinoma, endometrioid carcinoma, and clear cell carcinoma. MLA is considered to be a Mullerian duct-derived tumor.^4^

Primary MLA of the uterine corpus accounts for approximately 1% of uterine corpus cancers. Patient ages range from 26 to 75 years, varying among studies. However, the median age at the time of diagnosis is 55.9 to 58 years; many studies reported its onset in postmenopausal women. In 50% of patients with primary MLA of the uterine corpus, a diagnosis was made at an advanced stage, FIGO stage III or IV. Diagnosis at an advanced stage is significantly more frequent than in patients with low-malignant endometrioid carcinoma of the uterine corpus (approximately 18%).^5^ Furthermore, MLA more frequently develops at a young age compared with other histological types of high-grade uterine corpus cancer, and 1/2 or greater muscle layer infiltration and vascular invasion are more frequent.^5,6^ Symptoms include genital bleeding, vaginal discharge, abdominal pain, and abdominal discomfort, as reported for other histological types of uterine corpus cancer. However, MLA is asymptomatic in some cases.^5,7,8^ Histologically, intracytoplasmic mucus-free, low- to intermediate-grade cubiform or columnar cells proliferate by forming a tube containing eosinophilic hyaline-like secretion, imitating the mesonephric tube, as described for MA. However, proliferation patterns vary: fissure-like voids, papillary growth, and solid sheet-like/glomerular/luminal/cord-like/maze-like/microcyst-like patterns; therefore, MLA must be differentiated from endometrioid carcinoma, serous carcinoma, and uterine tumors resembling ovarian sex cord tumors.^2,9^ MLA histologically resembles MA. However, sites differ, and the mesonephric remnant or hyperplasia in the inner area of the tumor or at its periphery is observed in MA patients, but not in MLA patients. Based on these findings, differential diagnosis is performed.^6,10^

In the present case, a sarcomatous change was observed in addition to MLA. However, there are few such case reports. Park et al. reported 12 patients with endometrial MLA complicated by sarcoma. However, in most patients (10/12), MLA comprised ≥80% of the tissue, and the sarcomatous component comprised ≤20%.^11^

As primary MLA of the uterine corpus is a recently, newly defined tumor,^5^ it is not well known, being rare. In addition, its histological variety makes diagnosis extremely difficult. According to a report by Euscher et al., pathological consultation or referral to other hospitals was required for diagnosis in 20 of 23 patients diagnosed with MLA between 2004 and 2019. Only 3 were definitively diagnosed at their institution.^5^ Furthermore, preoperative biopsy-based diagnosis is difficult in many cases of MLA, as demonstrated in the present case. According to a study,^6^ other histological diagnoses were made on preoperative endometrial histodiagnosis in 5 of 7 patients finally diagnosed with MLA of the uterine corpus. A small amount of biopsy specimens reflect only a portion of various specific tumor backgrounds; diagnosis is difficult.^12^ In the present case, the tumor had reached the uterine cervix, and cervical biopsy was performed. However, the collected tissue volume was small, and only a portion of undifferentiated tumor features was collected, not leading to a preoperative diagnosis of MLA. The accurate diagnosis rate may be improved by increasing the biopsy specimen volume through full endometrial curettage under anesthesia.

Immunohistochemically, MLA also resembles MA. Tumor cells are positive for GATA3, TTF-1, CD10, and PAX-8, negative or partially positive for ER, and negative for PR and WT1.^2,6^ Of these, MLA is positive for ≥2 mesonephric markers, and immunostaining is useful for diagnosis.^6^ In the present case, tumor cells were also positive for GATA3, TTF-1, CD10, and PAX-8, and this finding was extremely useful for making a diagnosis.

Histologically, endometrial MLA must be initially differentiated from endometrioid carcinoma. However, if there are no characteristic findings of endometrioid carcinoma, such as endometrial hyperplasia as a background factor, tubular proliferation of tall columnar cells with the proliferative-phase-like dark cytoplasm, squamous epithelial differentiation, or mucous cell differentiation, MLA must also be considered in addition to endometrioid carcinoma for differential diagnosis.^5^ The above immunostaining findings and gynecologic pathology consultation are helpful for making a definitive diagnosis.^5,6,13^ From the viewpoint of molecular biology, MLA is associated with gene mutations. According to several studies, the KRAS mutation accounted for approximately 50%, the PTEN mutation for 35%, and the CTNNB1 mutation for 12%.^5,14,15^ In some patients, cancer genomic profiling tests may reveal mutations, being useful for selecting a treatment method.^14,16^

Concerning the prognosis, a study followed-up 21 patients with MLA of the uterine corpus, and reported relapse in 15 patients (71%), with a median progression-free survival (PFS) of 17 months (4 to 84 months).^5^ Of the 15 patients, the sites of relapse were the lungs in 9, liver in 2, peritoneum in 1, pelvis in 1, and vagina in 1. Pulmonary relapse was the most frequent.^5^ Other studies also reported that lung metastasis was the most frequent. However, the site of metastasis varies: the regional lymph nodes, liver, pancreas, and spleen. MLA is characterized by early distant metastasis.^5,6^ With respect to overall survival (OS) in addition to PFS, the prognosis of MLA patients is reportedly poorer than that of patients with high-grade uterine corpus cancer.^5^

Although the number of studies is further limited, the component of recurrent tumors that could be investigated was MLA, but not sarcoma, in ≥90% of MLA patients with the sarcomatous component, as demonstrated in our patient. MLA may contribute to the prognosis rather than the sarcomatous component.^11^

A consensus regarding treatment for MLA of the uterine corpus has not been reached. However, many studies performed hysterectomy/salpingo-oophorectomy and regional lymph node dissection as surgical techniques, radiotherapy, and chemotherapy in accordance with other histological types of uterine corpus cancer.^6^ A study reported a specific response to TC therapy,^17^ as described for other histological types of uterine corpus cancer. However, treatment resistance and early relapse are frequently detected, and the long-term prognosis is clearly poor. In the future, a larger number of case reports should be accumulated.

In the present case, follow-up was continued without performing postoperative chemotherapy, but multiple brain metastases were detected early after surgery. Thus, strict follow-up may be necessary, considering that early lung/brain metastases are frequent in MLA patients in comparison with common types of uterine corpus cancer.

## Conclusion

We encountered an extremely rare patient with primary MLA of the uterine corpus accompanied by the sarcomatous component. The malignancy level of MLA is higher than that of other histological types of uterine corpus cancer, and the prognosis is poor. However, neither treatment nor follow-up strategies have been established. In the future, a larger number of patients should be accumulated for further research.

## Supplementary Material

PDF-Japanese
