# Supplementary material for: Mesonephric-like adenocarcinoma of the uterine corpus with focal sarcomatous differentiation: A case report
Source: Fujita Med J. 2024 Oct 31;11(1):54–8. doi: 10.20407/fmj.2024-017 (PMC11782939; doi:10.20407/fmj.2024-017)
Supplement: Supplementary file 1 — PDF-Japanese [file fmj-11-054-s001.pdf]

症例報告

子宮体部に発生し肉腫様分化を伴った中腎様腺癌 Mesonephric-like adenocarcinoma の 1 例

Mesonephric-like adenocarcinoma with focal sarcomatous differentiation of the uterine corpus ; A case report

ランニングタイトル：肉腫様分化を認めた子宮体部の中腎様腺癌

藤田医科大学ばんたね病院 産婦人科 [1)]、同　病理診断科 [2)]
[1)] Department of Obstetrics and Gynecology, Fujita Health University Bantane Hospital, Nagoya, Aichi, Japan
[2)] Department of Diagnostic Pathology, Fujita Health University Bantane Hospital, Nagoya, Aichi, Japan
金尾　世里加 [1)]、浦野　誠 [2)]、藤田　和寿 [1)]、内海　史 [1)]、杉原　一廣 [1)]、柴田　清住 [1)]
Serika KANAO,MD, Makoto URANO,MD,PhD, Kazuhisa Fujita MD, Fumi UTSUMI,MD,PhD, Kazuhiro SUGIHARA,MD,PhD, Kiyosumi SHIBATA,MD,PhD.

金尾　世里加, MD
Department of Obstetrics and Gynecology, Fujita Health University Bantane Hospital,
3-6-10, Otobashi, Nakagawa-ku, Nagoya, Aichi 454-8509, Japan
052-321-8171
celica1104@gmail.com

【要旨】

子宮体部の中腎様腺癌は 2016 年に初めて報告され、2020 年 WHO 分類第 5 版に新たな項目として追加された。子宮体癌の約 1%の頻度と少なく、その多彩な組織像のため病理診断が困難であるが、他の組織型の子宮体癌と比較し進行期で診断され悪性度が高く予後不良と報告されている。さらに子宮体部の中腎様腺癌に肉腫が混在する症例は報告が少ない。今回われわれは子宮頚癌と判断し術前化学療法を実施したが術後の摘出標本により肉腫成分が混在する体部発生の中腎様腺癌と診断された症例を経験したため報告する。

Key Words：中腎様腺癌、子宮体癌、肉腫

【症例】

患者：65 才、0 妊 0 産、閉経 50 歳

現病歴：自覚症状なし。近医で健康診断を受診したところ胸部レントゲン検査で肺野に多発結節影を指摘された。精査目的に胸部-骨盤 CT 検査を実施したところ子宮腫大および肺野の多発腫瘍を認め、子宮原発の悪性腫瘍を疑われ精査目的に紹介となった。

既往歴：良性甲状腺腫（20 代で手術）、高血圧

家族歴：特記事項なし

診察時所見:子宮頚部は肉眼的に腫瘍に置換されており内診で子宮は成人頭大、可動性は不良で左側の傍組織浸潤が疑われた。

骨盤造影 MRI：子宮頚部から体部にかけて腫瘍を認め腫瘍サイズは約 15×8.5×10cm 大であった。腫瘍は T1 強調像・T2 強調像と

もに不均一な低信号を呈し内部に一部出血像を認め、前壁側では漿膜を超える浸潤が疑われた。(図 1-A)

胸部-骨盤造影 CT：多発肺転移（図 1-B)、その他遠隔転移なし

腫瘍マーカーは SCC： 0.7 ng/ml、CA125 30.3 U/ml、CA19-9 2.6 U/ml、CEA<1.7ng/mL といずれも正常範囲であった。

子宮頸部組織診：表層の被覆重層扁平上皮には異型は認めなかったが深層で小型の集塊状から散在性に分布する N/C 比の高い裸核状の異型細胞からなる腫瘍胞巣が多数認められた（図 2-A)。腫瘍細胞は粗いクロマチンパターンを呈しており（図 2-B)、免疫染色で p16 陰性ではあったが、synaptophysin が部分的に陽性であったことから小細胞神経内分泌癌の可能性を疑った。

子宮内膜細胞診：子宮頸部組織検体と同様の N/C 比の高い裸核状細胞集団を認めた（図 2-C)。

腫瘍が子宮頸部から体部全体に及んでいたため原発部位の特定は困難であったが、小細胞神経内分泌癌とすると頸部原発の可能性が高いと考え、治療開始時は子宮頸癌 IVB 期と診断し化学療法を実施した。

治療経過：TC 療法（PTX：175mg/m_2、CBDCA：AUC6）を合計 3 コース実施したところ CT 検査で原発巣および多発肺陰影のいずれも縮小傾向を認め PR と判断した。TC 療法を続行し合計 7 コース実施したところ、CT 検査で原発巣は著明に縮小し多発肺病変はいずれも消失し同定不能となった。画像所見から腫瘍縮小が確認され内診所見からも外科的摘出が可能と判断され、生検の組織診断が小細胞神経内分泌腫瘍であったことも考慮して開腹手術を実施した。

手術所見：全身麻酔下に下腹部正中切開で開腹、子宮は手拳大ほどのサイズであり両側付属器は肉眼的に正常であった。腹水はほぼ認めず。治療前の画像では膀胱子宮窩腹膜への浸潤所見があったが、同部は瘢痕化しており癒着を認めるも剥離可能であった。その他腹腔内に明らかな腫瘍の残存病変なく準広汎子宮全摘、両側付属器摘出術を実施。左閉鎖リンパ節にわずかな腫大を認め生検目的に摘出した。手術時間 2 時間 35 分、出血量 200ml であり術中合併症なく終了した。子宮径は 5×8×3cm であった。腫瘍径は 4×4cm で子宮頸部から体部に連続し位置していた（図 3）。

手術材料の組織像：腫瘍部分は類円形～多角形核細胞が好酸性物質を含む融合腺管状（図 4-A)、裂隙状～不整形腺管状（図 4-B)、まばらな腺管構造を含む索状（図 4-C）あるいは充実性胞巣状構造（図 4-D）からなる多彩な癌腫像を呈していた。また部分的に異型を伴う紡錘形異型細胞が束状に増殖する肉腫様の領域を認めた（図 5)。背景に既存の内膜腺の残存は明らかでなかった。免疫染色では腫瘍細胞は GATA3(図 6-A)、CD10(図 6-B)、calretinin、TTF-1、Pax-8 に種々の程度に陽性、ER は陰性であった。

以上の所見から、背景に中腎管の遺残は確認されず、術前の子宮頸部生検検体で被覆扁平上皮に悪性所見を認めず上皮下に腫瘍発育があったこと、摘出標本において腫瘍の肉眼的・組織学的な広がりは子宮頸部よりも子宮体部側の内膜が主体であったことから、病変の主座は子宮体部である可能性が高いと考えられ子宮体部原発の中腎様腺癌と診断した。

また、腫瘍の一部にみられた紡錘形細胞の増殖する領域では GATA3 発現の低下、消失傾向が認められ sarcomatous

differentiationと判断した（図5)。また、生検検体で小細胞神経内分泌癌が疑われた像と類似するN/C比の高い裸核状細胞の充実性増殖部分は、摘出組織では頸部側を主体に観察され、synaptophysinの軽度発現がみられたことから、本腫瘍の低分化な成分をみていたものと考えられた。摘出リンパ節検体に転移は認めなかった。また、術前の画像検査では化学療法によって腫瘍の縮小を認めていたが、摘出標本の腫瘍細胞に変性は乏しく、化学療法に対する抵抗性が出現していることが示唆された。上記の病理結果から最終診断は子宮体癌（中腎様腺癌）ypT2N0M1, Stage IVBとなった。術後経過は順調で術後12日目に退院した。追加治療を希望せず術後補助療法を行わず経過観察を行う方針とし外来受診していたが、術後約17週後に呂律障害と歩行困難を自覚し救急搬送された。造影脳MRIを撮影するとT2強調画像で両側大脳半球白質に嚢胞を伴う高信号域の腫瘤が多発しており多発転移が疑われた（図7)。全身麻酔下で脳病変を生検したところ、前述の子宮腫瘍と同様の所見を認めたため転移と判断し現在γナイフによる治療中である。

【考察】

中腎腺癌 mesonephric adenocarcinoma(MA)は中腎管の遺残により発生すると考えられている一方、中腎腺癌の組織学的特徴を持ち構造は類似しているものの背景に中腎管の遺残や過形成が確認できないものを中腎様腺癌 mesonephric-like adenocarcinoma（MLA）という[1,2]。子宮体部の中腎様腺癌は2016年に初めて報告され、その後2020年のWHO分類第5版に子宮体部腫瘍として追加された[1、2、3]。

中腎様腺癌は子宮体部および卵巣に発生し多様な増殖パターンが混在していることが特徴で内腔に好酸性分泌物を含み、時に甲状腺乳頭癌の核に類似する特徴的な核を有する腫瘍と定義される[1]。中腎腺癌では中腎管（ウォルフ管）の遺残により発生すると考えられているが、中腎様腺癌では中腎管の遺残を欠きながらも中腎腺癌とほぼ同様の組織学的特徴をもつためその発生母地に関してはこれまで議論されてきた。現在では、子宮内膜症や境界悪性腫瘍、低分化漿液性癌、類内膜癌、明細胞癌など Müllerian tumor をしばしば合併することがありミュラー管由来の腫瘍と考えられている[4]。

子宮体部原発の中腎様腺癌は子宮体癌全体の中で約１％の頻度とされ、患者の年齢は 26 歳から 75 歳と報告に幅があるが診断時の年齢の中央値は 55.9～58 歳と閉経後の報告が多い。子宮体部原発の中腎様腺癌の 50％が FIGO stage III もしくは IV の進行期で診断されており、低悪性度の類内膜癌の子宮体癌が約 18％であったのと比較し有意に進行期での診断が多い[5]。また、中腎様腺癌においては他の組織型の高異型度子宮体癌と比較して若年発症が多く、1/2 以上の筋層浸潤や脈管侵襲の頻度が高い[5,6]。症状は他の組織型の子宮体癌と同様に性器出血や帯下異常、腹痛や腹部不快感などであるが、無症状であることもある[5,7,8]。組織学的には中腎腺癌と同様に、中腎管を模倣し細胞質内粘液を欠く低～中異型度の立方状ないし円柱状の細胞が好酸性の硝子様分泌物を含む管腔を形成して増殖するが、裂隙状の空隙や乳頭状発育、充実性シート状、糸球体様、管腔状、索状、迷路状、小嚢胞状など多彩な増殖パターンがみられることから、類内膜癌、漿液

性癌、卵巣性索腫瘍に類似した子宮腫瘍等との鑑別を要する[2,9]。中腎様腺癌では中腎腺癌と組織学的に類似しているが発生部位が異なることと中腎腺癌では腫瘍の内部やその周囲に中腎管の遺残や過形成が観察されるが中腎様癌ではみられないことなどから鑑別診断する[6,10]。
また、本症例においては中腎様腺癌とともに肉腫様変化が観察されたが、このような症例の報告は少ない。Park らは子宮内膜の中腎様腺癌に肉腫を合併した 12 例の報告をしているが、組織の割合は 80%以上が中腎様腺癌であり、肉腫成分が 20%以下である症例が多数（10/12 例）を占めていた[11]。

子宮体部原発の中腎様腺癌は近年新しく定義された腫瘍であるため[5]十分に周知されておらず、希少な腫瘍であることに加え組織学的多様性により診断が極めて難しい。Euscher らの報告では、2004 年から 2019 年の間で中腎様腺癌と診断された 23 例のうち、診断に病理学的コンサルトや他院への紹介を要した症例が 20 例で自施設での確定診断例はわずか 3 例であったという[5]。また、中腎様腺癌は本症例のように術前の生検診断が困難なことが多く、子宮体部の中腎様腺癌と最終診断された 7 例のうち 5 例では術前の子宮内膜組織診では他の組織診断がされていた報告があり[6]、少量の生検検体では特有の多彩な腫瘍背景の一部しか反映できず診断が難しい[12]。本症例においても子宮頸部まで至る腫瘍であり頸部生検を行ったが、採取組織が少なく未分化な腫瘍像の一部のみの採取であったために中腎様腺癌の術前診断に至ることができなかった。麻酔下で子宮内膜全面掻爬を行うなど、生検において採取組織量を増やすことにより正診率が向上する可

能性があると推測される。
また、免疫組織化学的にも中腎腺癌と類似しておりGATA3、TTF-1、CD10、PAX-8が陽性でERは陰性もしくは部分陽性、PR陰性、WT1陰性を示すと言われる[2,6]。このうち中腎様腺癌では2つ以上の中腎性マーカーが陽性になるとの報告があり免疫染色は診断に有効である[6]。本症例においてもGATA3、TTF-1、CD10、Pax-8が陽性であり、診断に至る上で極めて有用な所見であった。
組織学的に子宮内膜の中腎様腺癌はまず類内膜癌と鑑別する必要があるが、増殖症を背景とし増殖期類似の暗調な胞体を有する高円柱状細胞の管状増殖がみられ扁平上皮分化や粘液細胞の分化を伴うという類内膜癌の特徴的な所見がない場合は類内膜癌だけでなく中腎様腺癌も鑑別診断に入れる必要がある[5]。確定診断には前述の免疫染色の所見や婦人科病理コンサルトが一助となる[5,6,13]。また、分子生物学的に中腎様腺癌は遺伝子変異と関連しており、*KRAS*変異が約50%、*PTEN* 35%、*CTNNB1* 12%との報告があり[5,14,15]症例によってはがんゲノム検査を実施すると変異が見つかり治療法の選択に役立つ可能性がある[14,16]。
予後に関しては子宮体部の中腎様腺癌 21 例を経過観察した報告によると、15例（71%）で再発を認めPFSの中央値は17ヶ月（4-84ヶ月）であった[5]。この15例の再発例のうち、再発部位は肺9例、肝臓2例、腹膜や骨盤、膣がそれぞれ1例であり肺再発が最も多く報告されている[5]。他の報告でも肺が転移部位として最も多く報告されているが、所属リンパ節、肝臓、膵臓、脾臓など転移部位は多岐にわたり、早期から遠隔転移が観察されることが特徴的である[5,6]。また、PFSだけでなくOSに関しても中腎様

腺癌は高異型度の子宮体癌と比較し予後が不良であると報告されている[5]。

さらに報告が少ないが、本症例のように中腎様腺癌に肉腫成分が混在する症例では、調査可能であった再発腫瘍の成分は 90%以上が肉腫でなく中腎様腺癌が検出されており肉腫成分よりも中腎様腺癌が予後に寄与する可能性がある[11]。

子宮体部の中腎様腺癌に対する治療として統一された見解はないものの、他の組織型の子宮体癌に準じ手術として子宮・付属器摘出および所属リンパ節の摘出や放射線治療、化学療法などを実施する報告が多い[6]。他の組織型の子宮体癌同様に TC 療法が一定の奏功を認める報告もあるが[17]、治療抵抗性や早期からの再発も多く明らかに長期予後は不良であり今後さらなる症例の報告が待たれる。

本症例においても術後化学療法を実施せず経過観察を行うも術後早期に多発脳転移を認めた。このように中腎様腺癌では通常の子宮体癌と比較し早期の肺・脳転移などへの再発が多いことに留意し厳重なフォローアップが必要であると考える。

【結語】

子宮体部原発の中腎様腺癌に肉腫成分が混在する極めてまれな症例を経験した。中腎様腺癌は他の組織型の子宮体癌と比較して悪性度が高く予後不良であるが、その治療方針や経過観察に関する方針はまだ示されておらず今後の症例蓄積と研究が待たれる。

【謝辞】

本例の病理診断につきご教示を頂きました滋賀医科大学病理部病理診断科 森谷鈴子先生に御礼申しあげます。

【利益相反】

本論文に関わる著者の利益相反：なし

文献

1. McFarland M, Quick CM, McCluggage WG. Hormone receptor-negative, thyroid transcription factor 1-positive uterine and ovarian adenocarcinomas: report of a series of mesonephric-like adenocarcinomas. Histopathology. 2016;68:1013-20.

2. Japan Society of Obstetrcis and Gynecology, The Japanese Society of Pathology. The general rules for clinical and pathological management of uterine corpus cancer. Pathological edition . 5th ed Tokyo: Kanehara; 2022: 37,68-9(in Japanese).

3. McCluggage WG, Singh N, Gilks CB. Key changes to the World Health Organization (WHO) classification of female genital tumours introduced in the 5th edition (2020). Histopathology 2022;80:762-78.

4. Mirkovic J, Olkhov-Mitsel E, Amemiya Y, Al-Hussaini M, Nofech-Mozes S, Djordjevic B, Kupets R, Seth A, McCluggage WG. Mesonephric-like adenocarcinoma of the female genital tract: novel observations and detailed molecular characterisation of mixed tumours and mesonephric-like carcinosarcomas. Histopathology 2023;82:978-90.

5. Euscher ED, Bassett R, Duose DY, Lan C, Wistuba I, Ramondetta L, Ramalingam P, Malpica A. Mesonephric-like Carcinoma of the Endometrium: A Subset of Endometrial Carcinoma With an Aggressive Behavior. Am J Surg Pathol 2020;44:429-43.

6. Kim HG, Kim H, Yeo MK, Won KY, Kim YS, Han GH, Kim HS, Na K. Mesonephric-like Adenocarcinoma of the Uterine Corpus: Comprehensive Analyses of Clinicopathological, Molecular, and Prognostic Characteristics With Retrospective Review of 237 Endometrial Carcinoma Cases. Cancer Genomics Proteomics 2022;19:526-39.

7. Euscher ED, Marques-Piubelli ML, Ramalingam P, Wistuba I, Lawson BC, Frumovitz M, Malpica A. Extrauterine Mesonephric-like Carcinoma: A Comprehensive Single Institution Study of 33 Cases. Am J Surg Pathol 2023;47:635-48.

8. Xie C, Chen Q, Shen Y. Mesonephric adenocarcinomas in female genital tract: A case series. Medicine (Baltimore) 2021;100:e27174.

9. Pors J, Segura S, Chiu DS, Almadani N, Ren H, Fix DJ, Howitt BE, Kolin D, McCluggage WG, Mirkovic J, Gilks B, Park KJ, Hoang L. Clinicopathologic Characteristics of Mesonephric Adenocarcinomas and Mesonephric-like Adenocarcinomas in the Gynecologic Tract: A Multi-institutional Study. Am J Surg Pathol 2021;45:498-506.

10. Tanaka R, Kimura K, Nishi S, Ikeda K, Wakui Y, Kikuchi H. A case of ovarian mesonephric-adenocarcinoma diagnosed after laparoscopic surgery. The Medical Journal of KKR Sapporo Medical Center 2021;18:46-9(in Japanese).

11. Park S, Park E, Kim HS. Mesonephric-like Carcinosarcoma of the Uterine Corpus: Clinicopathological, Molecular and Prognostic Characteristics in Comparison With Uterine Mesonephric-like Adenocarcinoma and Conventional Endometrial Carcinosarcoma. Cancer

Genomics Proteomics 2022;19:747-60.

12. Seo Y, Park E, Kim HS. Cytological features of mesonephric-like adenocarcinoma of the uterine corpus. Diagn Cytopathol 2023;51:294-306.

13. Kamihara Y, Takeshita S, Hayashi M, Sassa H. Mesonephric-like carcinoma of the uterine corpus: a case report. Japanese Journal of Gynecological Oncology 2019;37:741-7(in Japanese).

14. Brambs CE, Horn LC, Hiller R, Krücken I, Braun C, Christmann C, Monecke A, Hohn AK. Mesonephric-like adenocarcinoma of the female genital tract: possible role of KRAS-targeted treatment-detailed molecular analysis of a case series and review of the literature for targetable somatic KRAS-mutations. J Cancer Res Clin Oncol 2023; 149: 15727-36.

15. Yasaka M, Ikeda M, Machida H, Iida T, Shida M, Yoshida H, Hirasawa T, Kajiwara H, Mikami M. Five cases of mesonephric-like carcinoma of female genital tract. Japanese Journal of Gynecologic Oncology 2021;39:648-55(in Japanese).

16. Kajiwara H, Hirasara T, Kawashima M, Matsui N, Mikami M, Osamura R, Nakamura N. Three cases of mesonephric-like adenocarcinoma in the female genital organs. Obstetrics and Gynecology Reports. 2020;4.

17. Deolet E, Van Dorpe J, Van de Vijver K. Mesonephric-Like Adenocarcinoma of the Endometrium: Diagnostic Advances to Spot This Wolf in Sheep's Clothing. A Review of the Literature. J Clin Med 2021;10:698.

図のキャプション

図 1-A

治療前の骨盤単純 MRI 画像(T2 強調画像)：子宮頸部から体部を占拠する低信号の腫瘍を認めた。腫瘍内部には出血像を認め、前壁側では漿膜を超える浸潤が疑われた。

1- B　胸部 CT 画像:両側肺野に多発転移を疑う腫瘤像を認めた。

図 2　術前病理所見

2-A：頸部生検（HE 染色、×4）

被覆重層扁平上皮には異型は認めず、深層に小集塊状から散在性に腫瘍胞巣が多数認められた。

2-B：頸部生検（HE 染色、×20）

N/C 比大で核クロマチンが粗く挫滅を伴う裸核状細胞が胞巣状、充実性シート状増殖を呈していた。

2-C：内膜細胞診（Papanicolaou 染色、×40）

子宮頸部検体と同様、N/C 比の高い裸核状細胞の重積性集塊の出現を認めた。

図 3　摘出子宮および両側付属器の肉眼像

　頸部から体部にかけて約 4×4cm 大の腫瘍を認めた。

図 4　摘出子宮の病理組織像

4-A：好酸性物質を含む融合腺管状増殖像（HE 染色、×20）

4-B：裂隙状～不整形腺管状増殖像（HE 染色、×20）

4-C：索状増殖像（HE 染色、×20）

4-D：充実性胞巣状増殖像（HE 染色、20）

図 5　摘出子宮の病理組織像（HE 染色、×20）

異型紡錘形細胞の束状増殖からなる肉腫様部分像

図 6　免疫染色像

6-A：GATA3 に陽性（×20）

6-B：CD10 に陽性（×20）

図 7　脳 MRI　T2 強調画像で両大脳半球に嚢胞を伴う高信号の病変を認める。
